# Japanese version of the motivation to change lifestyle and health behaviors for dementia risk reduction scale: a cross-cultural validation

**DOI:** 10.3389/ijph.2026.1609357

**Published:** 2026-05-07

**Authors:** Fumiya Nakai, Toshiro Horigome, Mayu Fujikawa, Haruaki Horie, Masaru Mimura, Taishiro Kishimoto

**Affiliations:** 1 Center for the Promotion of Interdisciplinary Research in Medicine and Life Science, Keio University School of Medicine, Tokyo, Japan; 2 Hills Joint Research Laboratory for Future Preventive Medicine and Wellness, Keio University School of Medicine, Tokyo, Japan; 3 Department of Neuropsychiatry, Keio University School of Medicine, Tokyo, Japan; 4 Center of Preventive Medicine, Keio University, Tokyo, Japan

**Keywords:** dementia, health behavior, lifestyle change, motivation, questionnaire

## Abstract

**Objectives:**

This study examined the reliability and validity of a Japanese version of the Motivation to Change Lifestyle and Health Behaviors for Dementia Risk Reduction (MCLHB-DRR) scale, which assesses the motivation to change lifestyle and health behavior for preventing major neurocognitive disorder based on the health belief model.

**Methods:**

The scale was translated using Brislin’s translation model. A total of 500 Japanese adults aged 40–89 years were recruited online. The translated version was evaluated for content validity, internal consistency, and test-retest reliability over 2 weeks.

**Results:**

Exploratory factor analysis revealed a seven-factor model that explained 58% of the variance. Cronbach’s α was >0.7 for all seven subscales and item–total correlations were significant for all items. Confirmatory factor analysis exhibited a moderate fit, and the factor loadings were significant for all items. ICC (1,1) showed moderate test-retest reliability.

**Conclusion:**

The Japanese version of the MCLHB-DRR test showed high reliability and validity among Japanese older adults.

## Introduction

Japan, one of the world’s most advanced super-aged societies, in which the proportion of older adults (aged 65 and above) is expected to exceed 30% by 2025 [1–3], faces a significant challenge in addressing the increase in patients with major neurocognitive disorder (i.e., dementia). Therefore, preventive policies for major neurocognitive disorder are urgently needed. Because multiple risk factors for major neurocognitive disorder have been identified, multi-domain interventions are considered more effective than individual interventions targeting a single domain [4]. For instance, 14 lifestyle factors contributing to the risk of major neurocognitive disorder have been identified, including education, hypertension, obesity, hearing loss, traumatic brain injury, alcohol misuse, smoking, depression, physical inactivity, social isolation, diabetes, air pollution, high LDL cholesterol, and vision loss. Comprehensive solutions addressing these factors could potentially prevent or delay up to 45% of dementia cases [5].

Furthermore, the life stage during which these factors contribute to the risk of major neurocognitive disorder varies [6, 7]. For example, social isolation, air pollution, and vision loss have emerged as strong risk factors in later life. In contrast, factors such as hearing loss, high LDL cholesterol level, traumatic brain injury, depression, and physical inactivity play a more prominent role in middle life. In addition, low educational attainment has been identified as a strong risk factor for major neurocognitive disorder in early life [5]. These variations indicate that the risk of major neurocognitive disorder exists throughout a person’s lifetime. Therefore, rather than implementing numerous policies focused on individual risk factors, fostering the motivation to change lifestyle and health behavior for preventing major neurocognitive disorder at the individual level and encouraging diverse and comprehensive preventive behaviors throughout life may be a more effective strategy for the prevention of major neurocognitive disorder nationwide.

Research on disease prevention awareness has been conducted using various models, including the health belief model (HBM) [8], transtheoretical model [9], and social cognitive theory [10]. Among these, the HBM has been particularly effective in predicting future preventive behaviors [11]. Prevention awareness tests based on the HBM have been developed for several diseases, such as breast cancer [12, 13], cervical cancer [14, 15], and colorectal cancer [16, 17], showing correlations with participation rates for the screening of each disease. The HBM comprises four core components: perceived susceptibility, severity, benefits, and barriers. Additional variables, such as the overall motivation to pursue healthy behaviors [18], self-efficacy [19], and intention [20], have been proposed to expand the applicability of the model.

The Motivation to Change Lifestyle and Health Behaviors for Dementia Risk Reduction (MCLHB-DRR) scale [21] is a test assessing the motivation to change lifestyle and health behavior for preventing major neurocognitive disorder, based on the HBM (Table 1). This test was developed through a focus group interview involving 34 middle-aged and older Australian participants [22], combined with a review of existing HBM-based literature related to breast cancer screening [12] and cognitive state assessments [23]. Based on these, 53 initial questions addressing attitudes and beliefs surrounding health and lifestyle behavior and dementia risk were formulated. These questions were refined to 27 items through reliability and validity evaluations, through an online survey involving 659 Australian participants. The reliability and validity of this test were confirmed through confirmatory factor analysis, Cronbach’s α, item–total correlations, and test-retest reliability. Additionally, the scale was translated and validated in various languages, including Chinese [24], Turkish [25], Hebrew [26], and Dutch [27].

**TABLE 1:** Characteristics of study participants. (Japan, 2026).

N	​	1st	Retest
	​	500	401
Gender	Male	47.4%	50.1%
​	Female	52.6%	49.9%
Age	Male	59.6 ± 12.0	59.8 ± 11.8
​	Female	61.3 ± 12.6	61.8 ± 12.8
Education	Elementary school	1.0%	0.7%
​	Junior high school	3.0%	3.2%
​	High school	33.0%	32.4%
​	College or higher	63.0%	63.6%
Job	Retired or No job	22.6%	23.9%
​	Employee	28.6%	28.4%
​	Parttime	16.2%	14.7%
​	Self-employed	7.6%	8.0%
​	Housemaker	19.8%	19.0%
​	Others	5.2%	6.0%
Medical history	Diabetes	11.4%	12.5%
​	Hypertension	25.6%	23.9%
​	Hyperlipidemia	19.4%	18.7%
​	Cancer	8.4%	8.0%
​	Cerebro/Cardiovascular disorders	5.0%	5.5%
Dementia in family	​	13.2%	12.5%
Caregiving experience	​	23.4%	21.9%

However, studies focusing on prevention awareness of major neurocognitive disorder in Japan are limited; to the best of our knowledge, there is no test available in Japan to evaluate prevention awareness of major neurocognitive disorder. Therefore, this study aimed to develop a Japanese version of the test assessing the motivation to change lifestyle and health behavior for preventing major neurocognitive disorder based on the HBM, and evaluate its reliability and validity among healthy older adults in Japan. Enhancing individual awareness of prevention is a crucial approach for reducing the risk of major neurocognitive disorder. Thus, it is anticipated that the development of this test will contribute to future research on prevention of major neurocognitive disorder in Japan.

## Methods

### Questionnaire

The Japanese version of the motivation test to change lifestyle and health behavior for preventing major neurocognitive disorder was developed by translating the original English version of the MCLHB-DRR scale. This scale consists of 27 items derived from seven components of the HBM. They include the four core subscales—perceived susceptibility (four items), perceived severity (five items), perceived benefits (four items), and perceived barriers (four items)—as well as cues to action (four items), general health motivation (four items), and self-efficacy (two items). Each item is evaluated on a five-point Likert scale ranging from “strongly disagree” (score = 1) to “strongly agree” (score = 5).

### Development of the Japanese version of the MCLHB-DRR scale

The scale was translated following a revised version of the Brislin translation model. First, two researchers (NF and TK) proficient in both Japanese and English translated the original English version into Japanese. These translations were then integrated by multiple researchers (NF, TH, TK, and MM), including the two original translators. Subsequently, another researcher (MF) back-translated the integrated Japanese version into English. The researchers along with Ms. Sarang Kim, the original developer of the MCLHB-DRR test and two others (NF and TK) reviewed the back-translated English version and confirmed that there were no substantial differences from the original test.

### Participants and procedure

This study was approved by the Ethics Committee of Keio University School of Medicine. All participants provided informed consent by clicking the consent button after reading the study explanation on the website. Middle-aged and older adults (40–89 years) residing in Japan and proficient in Japanese were recruited online. Participants were recruited from a panel maintained by the survey company Cross Marketing Inc. A total of 500 participants were selected, to match the gender and age distribution of the Japan’s national population demographics in 2023 [28]. Participants received incentive points issued by the company as compensation. The survey was conducted online from November 2–3, 2024. Participants completed the Japanese version of the MCLHB-DRR test, along with background information (gender, age, occupation, and years of education). To assess test-retest reliability, the same participants completed the survey two to three weeks later.

### Statistical analyses

An exploratory factor analysis (EFA) was conducted to assess the internal validity of the test in terms of capturing the health belief model constructs. Prior to EFA, the Kaiser–Meyer–Olkin (KMO) coefficient was computed to determine the suitability of the data for factor analysis, and Bartlett’s test of sphericity was used to verify the robustness of the factor analysis. A KMO value of >0.6 was considered acceptable for sampling adequacy [29], and a significant Bartlett’s test (p < 0.05) indicated that factor analysis was appropriate. EFA was performed using the maximum likelihood estimation with oblique rotation. The number of factors was set to seven, corresponding to the original MCLHB-DRR subscales (see Supplementary Analysis). Following previous studies, items that had a loading <0.3 with their respective factors were removed [30].

Cronbach’s alpha and item–total correlations were calculated to assess the internal consistency of each subscale. Cronbach’s alpha was computed for each original subscale, and following the precedent, subscales with alpha values below 0.70 were considered unacceptable and items were removed [31]. In the item–total correlation analysis, items with values below 0.30 were excluded [32].

To evaluate the internal validity of the entire test, a confirmatory factor analysis (CFA) was performed using maximum likelihood estimation. To compare the internal validity with previous studies, the following five indices were examined: χ^2^/df, Comparative Fit Index (CFI), Goodness of Fit Index (GFI), Tucker-Lewis Index (TLI), and Root Mean Squared Error of Approximation (RMSEA). For χ^2^/df, values <3.0 were considered indicative of good fit and values <5.0 were considered indicative of reasonable fit [27, 33, 34]. Values of CFI and TLI >0.9 were considered indicative of adequate fit, while values >0.95 indicated good fit [33]. For RMSEA, values <0.08 considered reasonable error of approximation and <0.05 indicated close fit [35].

To assess the reliability of the test within participants, test-retest reliability was calculated. Specifically, for participants who completed both the initial and follow-up surveys, the intraclass correlation coefficient (ICC) was used to examine the between-participants consistency of the total scores for each subscale. ICC value was interpreted as follows: <0.5, poor; 0.5–0.75, moderate, 0.75–0.9, good; and >0.9 excellent [36].

All statistical analyses, except the test-retest reliability, were conducted using the results of the first survey. All analyses were performed using Python version 3.9.

## Results

The demographic characteristics of the participants are presented in Table 2. Five hundred participants (male: *n* = 237, mean age = 59.6 ± 12.0; female: *n* = 263, mean age = 61.3 ± 12.6) were included in the first survey; of them, 401 (80.2% of the initial sample; male: *n* = 201, mean age = 59.8 ± 11.8; female: *n* = 200, mean age = 61.8 ± 12.8) participated in the test-retest reliability survey. None of the participants were excluded due to missing responses, as all questions required a response.

**TABLE 2:** Item loadings of the exploratory factor analysis (seven-factor model) (Japan, 2026).

​	​	Factor 1	Factor 2	Factor 3	Factor 4	Factor 5	Factor 6	Factor 7
Perceived susceptibility	Q1	**0.897**	0.010	0.027	0.055	−0.033	−0.048	−0.019
​	Q2	**0.914**	0.015	−0.025	0.020	0.032	−0.024	−0.027
​	Q3	**0.915**	0.027	0.026	−0.025	0.031	0.018	0.018
​	Q4	**0.551**	−0.050	−0.006	−0.120	0.048	0.278	0.096
Perceived severity	Q5	0.173	0.162	−0.068	0.205	0.208	**0.304**	−0.163
​	Q6	0.089	−0.034	0.126	−0.032	−0.085	**0.727**	0.095
​	Q7	−0.007	0.176	−0.027	0.188	0.248	**0.533**	−0.232
​	Q8	0.025	−0.070	0.134	−0.109	−0.002	**0.736**	0.137
​	Q9	0.010	0.187	−0.008	0.150	0.194	**0.493**	−0.160
Perceived benefits	Q10	−0.009	**0.610**	−0.022	0.012	0.001	0.264	0.081
​	Q11	0.033	**0.927**	0.016	−0.054	−0.039	0.012	0.024
​	Q12	0.026	**0.810**	0.000	0.097	0.012	−0.059	0.009
​	Q13	0.012	**0.815**	0.005	0.024	0.065	−0.073	0.038
Perceived barriers	Q14	−0.069	0.126	**0.704**	−0.119	0.053	0.038	−0.001
​	Q15	0.101	0.014	**0.775**	0.041	0.003	−0.029	−0.095
​	Q16	0.039	−0.060	**0.844**	0.051	−0.024	0.068	0.045
​	Q17	−0.045	−0.006	**0.843**	0.010	0.041	−0.009	0.009
Cues to action	Q18	0.009	−0.023	0.136	0.057	**0.683**	0.054	−0.068
​	Q19	0.018	0.080	0.015	−0.030	**0.809**	−0.073	0.045
​	Q20	0.095	−0.048	−0.009	−0.021	**0.775**	0.039	0.124
​	Q21	0.027	0.034	−0.008	0.291	**0.509**	−0.032	0.015
General health motivation	Q22	−0.055	0.068	−0.002	**0.633**	0.112	0.009	0.077
​	Q23	0.066	−0.052	−0.063	**0.796**	−0.016	0.072	0.126
​	Q24	0.004	0.086	0.046	**0.840**	0.000	−0.110	0.048
​	Q25	0.106	0.035	0.076	**0.619**	0.087	0.050	−0.093
Self-efficacy	Q26	0.021	0.119	−0.066	0.167	0.112	0.066	**0.646**
​	Q27	−0.030	0.147	0.008	0.153	0.117	0.046	**0.651**

The KMO coefficient was 0.908. Bartlett’s test of sphericity showed significance (χ^2^ = 9,181, df = 351, p < 0.001). EFA conducted with seven factors explained 58.4% of the total variance and the first seven factors had eigenvalues greater than 1.0. The eigenvalue and the cumulative percentages of explained variance of the first seven factors were 2.87 (10.6%), 2.74 (20.8%), 2.59 (30.4%), 2.42 (39.4%), 2.19 (47.5%), 1.89 (54.5%) and 1.06 (58.4%). The factor loadings of each item on the detected seven-factor EFA model showed that all items loaded >0.3 on their respective factors, corresponding to the subscales in the original English scale (Table 2). No substantial cross-loadings (>0.3 on multiple factors) were observed and no items were deleted.

Cronbach’s α for each subscale was as follows: 0.904 (0.890–0.917) for perceived susceptibility; 0.792 (0.762–0.820) for perceived severity; 0.896 (0.881–0.910) for perceived benefits; 0.879 (0.860–0.895) for perceived barriers; 0.857 (0.835–0.876) for cues to action; 0.871 (0.851–0.888) for general health motivation; and 0.850 (0.822–0.874) for self-efficacy. The item–total correlations for all items ranged from 0.71 to 0.94 and were significant (Table 3, the corrected item-total correlation was also calculated. See Supplementary Table).

**TABLE 3:** Item–total correlations for all questionnaire items (Japan, 2026).

​	Item	Item-total correlation	*P*-value
Perceived susceptibility	Q1	0.908	*P* < 0.001
​	Q2	0.921	*P* < 0.001
​	Q3	0.938	*P* < 0.001
​	Q4	0.760	*P* < 0.001
Perceived severity	Q5	0.694	*P* < 0.001
​	Q6	0.738	*P* < 0.001
​	Q7	0.790	*P* < 0.001
​	Q8	0.717	*P* < 0.001
​	Q9	0.766	*P* < 0.001
Perceived benefits	Q10	0.778	*P* < 0.001
​	Q11	0.909	*P* < 0.001
​	Q12	0.907	*P* < 0.001
​	Q13	0.897	*P* < 0.001
Perceived barriers	Q14	0.808	*P* < 0.001
​	Q15	0.853	*P* < 0.001
​	Q16	0.888	*P* < 0.001
​	Q17	0.876	*P* < 0.001
Cues to action	Q18	0.812	*P* < 0.001
​	Q19	0.862	*P* < 0.001
​	Q20	0.864	*P* < 0.001
​	Q21	0.809	*P* < 0.001
General health motivation	Q22	0.835	*P* < 0.001
​	Q23	0.871	*P* < 0.001
​	Q24	0.896	*P* < 0.001
​	Q25	0.793	*P* < 0.001
Self-efficacy	Q26	0.933	*P* < 0.001
​	Q27	0.933	*P* < 0.001

CFA was performed to assess the internal validity of the entire test. The fit indices indicated a marginal fit (χ^2^/df = 4.086, CFI = 0.896, GFI = 0.868, TLI = 0.880, RMSEA = 0.0786). All the item factor loadings were statistically significant. All combinations of the inter-subscale correlations were significant, except for the following pairs: perceived benefits and perceived barriers, perceived barriers and general health motivation, and perceived barriers and self-efficacy.

The test–retest reliability was moderate, with an ICC (1,1) of 0.514.

## Discussion

In this study, we developed a 27-item questionnaire based on the HBM to assess the motivation to change the lifestyle and health behavior for preventing the neurocognitive disorder among Japanese participants by translating the MCLHB-DRR scale. To evaluate the reliability and validity of the Japanese version of the MCLHB-DRR scale, we conducted two surveys with 500 middle-aged and older adults and performed statistical analyses.

EFA revealed that a seven-factor model consistent with the original subscales explained 58.4% of total variance. All questionnaire items showed strong loadings exclusively with the factors corresponding to their respective subscales, indicating that the questionnaire adequately reflected the seven components of the HBM without any excesses or deficiencies. Additionally, Cronbach’s α and item–total correlation analyses showed that all items met the criteria, with no items rejected, confirming the high internal reliability of the questionnaire.

CFA further supported the internal validity of the questionnaire, as all items were significantly loaded with their respective subscales, and the model fit indices indicated a marginal fit. These findings suggest that the questionnaire appropriately represented the structure of the HBM. The results validated the development of the first Japanese test assessing the motivation to change lifestyle and health behavior for preventing major neurocognitive disorder based on the HBM, and were consistent with findings from previous studies conducted in other countries (Table 4).

**TABLE 4:** Previous studies using dementia prevention awareness tests based on the motivation to change lifestyle and health behaviors for dementia risk reduction scale (Japan, 2026).

Author and invested country	N. of sample	Min. age	RMSEA	CFI	GFI	TLI	χ^2^/df
Kim (2014, Australia)	617	50	0.047	0.92	0.92	Nan	2.38
Oliveira (2019, UK)	3,948	50	Na	Na	Na	Na	Na
Zehirlioglu (2019, Turkey)	220	40	0.061	0.88	0.84	Na	1.82
Akyol (2020, Turkey)	284	40	Na	Na	Na	Na	Na
Joxhorst (2020, Netherlands)	618	30	0.043	0.96	Na	0.95	2.13
Witbeck (2022, America)	477	18	Na	Na	Na	Na	Na
Lin (2023, China)	150	50	0.087	0.91	Na	Na	Na
Shvedko (2023, Israel)	328	50	0.072	0.87	Na	0.84	2.71
This study	500	45	0.079	0.90	0.87	0.88	4.09

Compared to previous studies that proposed the removal of some items from the original questionnaire, this study recommends retaining all 27 items. For instance, in the Chinese version, items 7 and 18 were removed; in the Hebrew version, items 6, 8, and 15 were removed; and in the Dutch version, items 4, 10, 13, and 25 were removed. Even when using the original English version, item 25 was recommended for removal in a North American study, while a United Kingdom study suggested using only 10 items. These inconsistencies in item removal across studies suggest that the importance of specific questionnaire items is strongly influenced by regional, linguistic, and sample-specific biases. It is also possible that these differences are influenced by national characteristics or ethnicity.

In this study, none of the items were recommended for removal. This consistency may reflect not only cultural differences between countries, but also similarities in recruitment methods between the present study and the interviews conducted in Australia, where the original questionnaire was developed. In the present study, participants were recruited through an online survey administered to individuals registered with a research panel. This approach is comparable to that used in the original Australian interviews, which also relied on recruitment through a survey company. In contrast, subsequent studies have employed different recruitment strategies, such as random sampling of residents (Netherlands), recruitment via social media (the United States, England, and Israel), and face-to-face interviews with users of elderly services (China). In fact, although the age and gender distributions of the present sample were carefully matched to Japan’s demographic statistics, the proportion of participants with higher education was substantially greater than that of the general Japanese population (37.6% in the general population [37]. This suggests that the participants may have been relatively accustomed to responding to questionnaires and may have had greater exposure to health-related information, which may have contributed to the observed consistency.

A limitation of this study is that it was conducted through an online survey, restricting the sample to middle-aged and older adults who regularly use the Internet. Consequently, individuals who do not use the Internet, possibly owing to socioeconomic reasons, were not included in this study. In addition, the present sample had a higher proportion of individuals with higher education compared to the general Japanese population. Socioeconomic status and educational background can be related to the prevention of major neurocognitive disorder, [5, 6]; therefore, future research should examine the generalizability of these findings. Furthermore, this study did not examine the external validity of the questionnaire, such as by comparing it with other established gold standard measures or by investigating its association with actual dementia prevention behaviors. One reason for this is the lack of well-established alternative indicators in this field. Therefore, the relationship between test results and future dementia prevention behaviors should also be investigated in future studies.

In conclusion, this study developed a Japanese version of a test assessing the motivation to change lifestyle and health behavior for preventing major neurocognitive disorder based on the seven components of the HBM, and confirmed its reliability and validity. All 27 items from the original MCLHB-DRR scale were retained without any exclusions. Future research should investigate the influence of individual characteristics on test results and examine effective policies to improve the motivation to change lifestyle and health behavior for preventing major neurocognitive disorder.

## Data Availability

The data that support the findings of this study are available from the corresponding author upon reasonable request.
