# Supplementary material for: Japanese version of the motivation to change lifestyle and health behaviors for dementia risk reduction scale: a cross-cultural validation
Source: Int J Public Health. 2026 May 7;71:1609357. doi: 10.3389/ijph.2026.1609357 (PMC13189982; doi:10.3389/ijph.2026.1609357)
Supplement: Supplementary file 1 [file Supplementaryfile1.docx]

**Supplementary analysis**

To examine the optimal number of factors, exploratory factor analysis was conducted specifying one to nine factors, and the corresponding AIC and BIC values were compared. The AIC values for the one- to nine-factor solutions were -24.6, -31.0, -34.6, -36.0, -39.3, -39.7, -38.9, -37.7 and -36.5, indicating that the six-factor solution was optimal. The BIC values for the one- to nine-factor solutions were -20.4, -22.6, -22.0, -19.1, -18.3, -14.4, -9.4, -4.0 and 1.5, indicating that the two-factor solution was optimal. However, solutions with fewer than six factors resulted in substantial cross-loadings (> 0.3) in several items and poor interpretability of the factor structure.

Additional confirmatory factor analysis was conducted for the eight-factor model. While the fit indices indicated an acceptable fit (χ²/df=2.87, CFI=0.939, GFI=0.910, TLI=0.927, RMSEA=0.0612), it appeared to overfactor the structure, leading to reduced conceptual clarity.

Taken together, these findings suggest that the seven-factor model represents the most appropriate balance between statistical fit and interpretability.

**Supplementary Table**

Corrected item–total correlations for all questionnaire items.

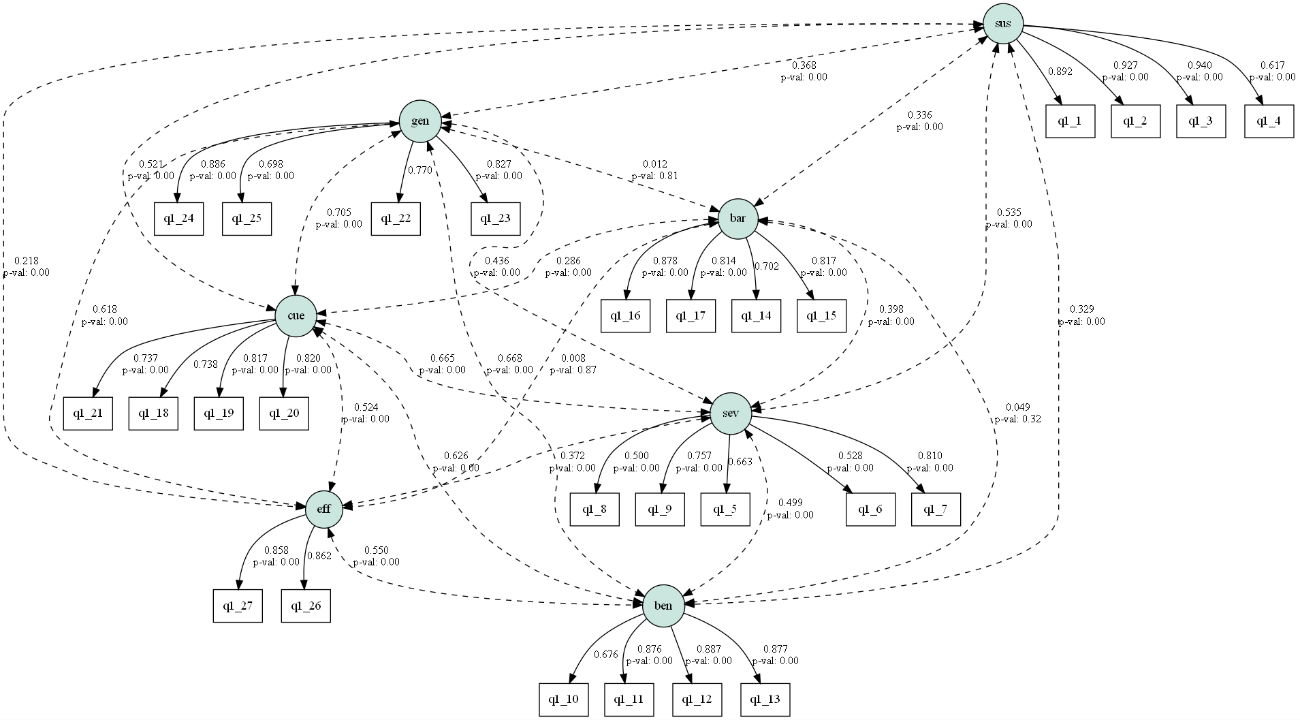


**Supplementary Figure.**

One-item-subscale and inter-subscale correlation of the confirmatory factor analysis model
