# Supplementary material for: Japanese version of the motivation to change lifestyle and health behaviors for dementia risk reduction scale: a cross-cultural validation
Source: Int J Public Health. 2026 May 7;71:1609357. doi: 10.3389/ijph.2026.1609357 (PMC13189982; doi:10.3389/ijph.2026.1609357)
Supplement: Supplementary file 2 [file DataSheet1.pdf]

認知症予防のための意識調査

お名前＿＿＿＿＿＿＿＿＿＿＿＿＿＿

あなた自身のことについて、お答えください

	全く思わない		どちらともいえない		とてもそう思う
Q1　自分が認知症になる可能性は高い	1	2	3	4	5
Q2　自分が将来認知症になる可能性は高いと感じる	1	2	3	4	5
Q3　自分が認知症になる可能性はかなり高い	1	2	3	4	5
Q4　今後10年以内に自分は認知症になるだろう	1	2	3	4	5
Q5　認知症のことを考えるのは怖い	1	2	3	4	5
Q6　認知症のことを考えると心臓の鼓動が速くなる	1	2	3	4	5
Q7　認知症になったら、自分に対する気持ちが変わってしまう	1	2	3	4	5
Q8　認知症のことを考えると吐き気がする	1	2	3	4	5

		全く<br>思わない		どちらとも<br>いえない		とても<br>そう思う
Q9	認知症になることは、他の病気になることよりも深刻だと思う	1	2	3	4	5
Q10	専門家からの情報提供やアドバイスにより、自分では考えもつかなかったことを知ることができ、認知症になる可能性が低くなるかもしれない	1	2	3	4	5
Q11	生活習慣や健康習慣を変えることは、自分が認知症になる可能性を減らすだろう	1	2	3	4	5
Q12	生活習慣や健康習慣を変えることで、自分が得るものは多い	1	2	3	4	5
Q13	より健康的な生活や行動に適応することは、私にとって認知症の予防になる	1	2	3	4	5
Q14	自分は忙しくて生活習慣や健康習慣を変える時間がない	1	2	3	4	5
Q15	自分は経済的な理由で生活習慣や行動を変えることができない	1	2	3	4	5
Q16	家庭の事情で、自分の生活習慣を変えることが難しい	1	2	3	4	5
Q17	生活習慣や行動を変えることは、私のスケジュールに支障をきたす	1	2	3	4	5

		全く<br>思わない		どちらとも<br>いえない		とても<br>そう思う
Q18	物忘れをすると、（予防のために）生活習慣や行動を変えなければならないと思う	1	2	3	4	5
Q19	認知症のリスクがあると、生活習慣や行動を変えなければならないと思う	1	2	3	4	5
Q20	メディアで認知症について知ると、生活習慣や行動を変えなければならないと思う	1	2	3	4	5
Q21	家族が認知症になったことを知ったら、生活習慣や行動を変えなければならないと思う	1	2	3	4	5
Q22	自分にとって健康以上に大切なものはない	1	2	3	4	5
Q23	自分の健康についてよく考える	1	2	3	4	5
Q24	自分の健康には気を配らなければならないと思う	1	2	3	4	5
Q25	自分の健康が心配だ	1	2	3	4	5
Q26	認知症になるリスクを減らすために、自分は生活習慣や行動を変えることができると確信している	1	2	3	4	5
Q27	自分は認知症になるリスクを変化させる行動を起こすことができる	1	2	3	4	5

以上で終わりです。ご協力ありがとうございました。
